# Factors associated with the use of digital health among healthcare workers in the Buea and Tiko health districts of Cameroon: a cross-sectional study

**DOI:** 10.11604/pamj.2024.47.51.35531

**Published:** 2024-02-08

**Authors:** Bill-Erich Nkongho Mbianyor Agboryah, Valirie Agbor Ndip, Armelle Viviane Ngomba, Alexis Awungia Tazinya, Dieudonné Adiogo

**Affiliations:** 1 Faculty of Medicine and Pharmaceutical Sciences, University of Douala, Douala, Cameroon,; 2 Jalpha Health Tech, Douala, Cameroon,; 3 Department of Population Health Research, Health Education and Research Organization, Buea, Cameroon,; 4 Clinical Trial Service Unit and Epidemiological Studies Unit, Nuffield Department of Population Health, University of Oxford, Oxford, United Kingdom,; 5 Division of Disease, Epidemics and Pandemics Control, Ministry of Public Health, Yaounde, Cameroon,; 6 Waspito Inc, Douala, Cameroon

**Keywords:** Digital health, healthcare workers, Buea, Tiko, health district, digital health intervention

## Abstract

**Introduction:**

digital health has been demonstrated to improve the efficiency and scale of health service delivery in resource-limited settings. Understanding factors influencing its use could accelerate the process of its implementation in routine practice.

**Methods:**

we conducted a cross-sectional analytic study in Buea and Tiko health districts from January to May 2021. We included healthcare workers selected using multistage stratified sampling. Use of digital health was defined as using at least two digital tools and one digital health intervention (DHI) or at least two DHIs by a healthcare worker. Epi Info was used for statistical analysis. Binary logistic regression was used to evaluate factors associated with the use of digital health.

**Results:**

in total, 221 participants were included in the study. The mean age was 33±9.1 years and 76.5% were female. Only 39.4% (n=87) of participants used digital health. The most frequently used digital tools for health-related purposes included: Microsoft (MS) Excel (29.9%), MS PowerPoint (26.8%) and MS Word (39.1%). The main DHIs used were research (30.2%) and diagnosing (24.1%) software. The main use of digital health was for research (75.6%). Owning a laptop (adjusted odds ratio (aOR)=1.98, 95% CI, 1.01 - 3.86), availability of internet connection in the health facility (1.99, 1.05 - 3.7) and receiving professional training in ICT/Computer Sciences (2.04, 1.06 - 3.93), were associated with higher odds of using digital health.

**Conclusion:**

this study shows a low level of use of digital health by healthcare workers. Providing newer devices, internet connection in health facilities and training in ICT for healthcare workers could improve its uptake.

## Introduction

Digital health comprises electronic health (eHealth) and mobile health (mHealth). eHealth is the cost-effective and secure use of information and communication technologies (ICTs) for health and health-related fields, including healthcare services, health surveillance, health literature, health education, knowledge and research. mHealth involves providing health services and information via mobile technologies such as mobile phones, tablet computers, and Personal Digital Assistants (PDAs) [1,2]. eHealth can significantly improve health service efficiency, expand or scale up treatment delivery to thousands of patients in developing countries, and improve patient outcomes [3].

Low-and-middle-income countries (LMICs) have increasingly transitioned from paper-based to digital information systems and are utilizing new technologies to collect data [4]. Though still in its early stages, mHealth has begun to transform health delivery throughout the developing world with concrete benefits, including increased access to healthcare and health-related information (particularly for hard-to-reach populations), improved ability to diagnose and track diseases and timelier and more actionable public health information [5]. The increasing uptake of digital technologies and data tools to support global health security allows countries to make more accurate and timely decisions for preventing, detecting and responding to outbreaks [6]. Data flow was a major hindrance to curbing the spread of the disease during the Ebola outbreak of 2014 that affected countries in West Africa such as Guinea, Liberia and Sierra Leone; only few data systems existed, which were weak and disorganized [7].

Despite significant advances in new digital tools and approaches, there has been inadequate capacity building of local and regional stakeholders. Without significantly increased investment in training, uptake of digital tools will be slow or misused. Health workers play a critical surveillance role in detecting disease outbreaks and their spread, and when empowered with digital technologies they could report these data in a timely way [7]. In Cameroon, the National Digital Health Strategic Plan (NDHSP) 2020 - 2024 released by the Ministry of Public Health (MOH) stated that most health staff and users, especially those in rural areas, do not have computer skills with many doctors and nurses primarily engaged in their technical work and consider ICT to be an additional burden that takes them away from their main tasks [8].

However, limited evidence exists on the use of digital technology by healthcare workers in routine clinical practice. Understanding determinants of use of digital technology among healthcare workers are vital in guiding policies to accelerate its implementation in routine practice. Therefore, this study seeks to understand the factors associated with the use of digital health among healthcare workers in two health districts in Cameroon.

## Methods

**Study design:** this was a hospital-based, cross-sectional, analytic study conducted from January to May 2021 in Buea and Tiko health districts of Cameroon.

**Study setting:** Buea and Tiko are two towns located in the South West Region of Cameroon. Buea is the administrative capital of the South West Region. It has a total population of about 59,765 inhabitants and is home to the country's first anglophone university and the only state-owned Faculty of Health Sciences in the Region [9]. The Buea Health District contains seven health areas namely Bokwango, Buea Town, Tole, Bova, Buea Road, Molyko and Muea health areas. There are 39 health facilities in the health district (including a regional hospital), with a higher ratio of private to public facilities.

Tiko is a semi-urban town and an important port in the South West region of Cameroon situated along the Bimbia River at the Gulf of Guinea. It has a surface area of 4840 square km and a population size of 48,220 inhabitants [9]. The Tiko Health District has eight health areas with 22 health facilities, including private health facilities such as the Cameroon Baptist Convention (CBC) Health Services and the CDC Cottage Hospital. These health areas include: Holforth, Kange, Likomba, Missellele, Mondoni, Mudeka, Mutengene and Tiko.

**Sample size estimation and sampling:** participants were selected using the multistage stratified approach. A list of all health facilities in each health district was obtained from the District Medical Office for a total of 63 health facilities in both districts. 15 of these health facilities were eliminated because political insecurity made them inaccessible leaving 48 health facilities remaining. Health facilities to be visited were randomly selected from health areas under each health district in proportion to the total number of health facilities under the health area. For health areas with ≤ 05 health facilities, we visited one third of the health facilities while for health areas with ≥ 05 health facilities, we visited half of the health facilities. A total number of 28 health facilities were visited. At least one-third of healthcare workers were selected from each facility by stratified sampling. No sample size calculation was done.

**Study population:** we included all healthcare workers currently working in selected health facilities of the Buea and Tiko health districts who consented to participate. Healthcare workers, not directly in contact with patients' clinical and paraclinical data and staff with purely administrative duties were excluded. In addition, healthcare workers, working in two or more health facilities in the same district were excluded from the study to avoid duplicate data.

### Study procedure

**Data collection:** data was collected using a structured questionnaire. A digital tool was defined as any software or application that enables the creation, management and sharing of information electronically [10]. They include MS Office, graphic design software such as Canva, and others. To examine the use of digital tools for health-related purposes, participants were asked if they used software such as MS Word, Excel or Canva for healthcare-related purposes, how often they used them and for what purposes. Examples of questions asked include “How often do you use MS Word for healthcare-related purposes such as recording and storing patient clinical data?” “Do you use graphic design software such as Canva to produce medical education content?” Digital Health Interventions as defined by WHO and used in this study are the different ways in which digital and mobile technologies are being used to support health system needs. They include patient health record systems, telemedicine services, healthcare provider research and training software, prescription and medication management and laboratory and diagnostic imaging management [11]. Participants were asked if they used digital health interventions available locally such as telemedicine apps, electronic health record software, medical research software, medical diagnostic software and e-prescribing software in their routine practice and how frequently they used them. Some examples of digital health interventions cited include software/applications such as Waspito for telemedicine, eClinic for electronic health records, SimplyBookMe for appointment scheduling, Human DX for medical diagnosing, and MedScape for research. Frequency of use was classified using the Likert scale from Never to Always.

**Variables:** the outcome variable using digital health was defined by any participant who made use of at least one digital health intervention and two digital tools for health-related purposes or at least two digital health interventions. Independent variables included: age, gender, type of facility, type of device owned, availability of internet connection within the health facility, receiving formal training in Information and Communication Technology (ICT), level of understanding of technology and perception of technology.

**Ethical and administrative approval:** this study was conducted following the declaration of Helsinki adopted in 1964. Ethical approval was obtained from the Institutional Ethics Committee of the University of Douala, decision number 2698IEC-UD/07/2021/T. Administrative clearance was then obtained from the South West Regional Delegation of Public Health, the Buea and Tiko Health District Medical Services and hospital included in the study. After detailed explanation of the purpose of the study. All participants signed an informed consent form before being included in the study. Participant's autonomy was respected and they were allowed to opt out of the study at any time. Participants benefitted from a brief education on digital health, its applications in low resource settings and digital health interventions available for use in their context.

**Data management and statistical analysis:** the data was entered and analysed using Epi Info Version 7. Microsoft Excel 2016 was used for data visualisation. Quantitative variables were described using means or medians while categorical variables were summarized using frequencies or percentages. The Chi-squared test was used to evaluate the significance of associations between predictor and outcome variables. Multivariable logistic regression analysis was used to identify factors independently associated with the use of digital health. All variables with a p < 0.2 after bivariate analysis were included in the multivariable logistic regression model. Any cases with missing data were omitted. The level of statistical significance was set at a p- value ≤ 0.05.

## Results

**Socio-demographic characteristics:** a total of 221 healthcare workers participated in the study with a mean age of 33 years (SD = 9.1). The majority of participants were female (76.5%), nurses (52.1%) worked in a private health facility (62%) and had between 1-5 years of work experience (40.6%).

**Availability and utility of digital devices:** the digital devices that were reported to be most available in facilities to participants were the fixed phone (51.6%), the desktop computer (51.6%) and the feature phone (44.3%). Most participants (90.5%) reported owning a smart phone and found it to be very/extremely (83.9%) useful for their routine practice. 80.9% of participants found the laptop to be very/extremely useful and 73.2% for the desktop. Stratified by profession, device utility for healthcare practice varied with doctors finding tablets to be more useful for digital health purposes while nurses preferred smartphones, laboratory technicians and pharmacy attendants preferred laptops.

**Figure 1:**
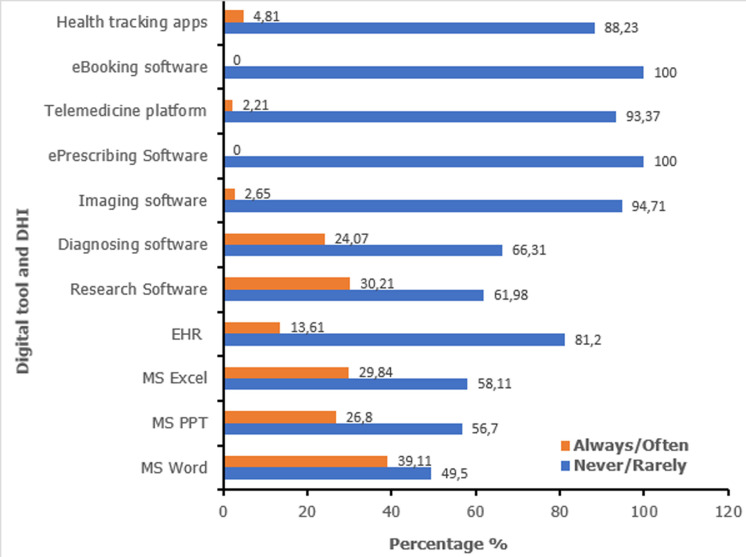
use of digital tools and digital health interventions

**Use of digital tools and DHI:** the majority of the participants (68.5%) had never used an electronic patient record (EPR) software while 13.7% reported having frequent access to EPR. Laboratory technicians were the highest proportions of participants who used EPR (1 in 2) while nurses comparatively were the lowest group with a ratio of 1 in 5. The use of digital tools for health-related purposes was low with only 29.8% of participants using MS Excel, 26.8% using MS PowerPoint and 39.11% of participants using MS word frequently. The use of DHI was very low. The main DHIs regularly used were research (30.2%) and diagnosing software (24.1%) while only 2.2% of participants made use of any telemedicine platforms and 2.7% of teleradiology (Figure 1). Other DHIs such as E-booking and E-prescribing were quasi-inexistent.

**Figure 2:**
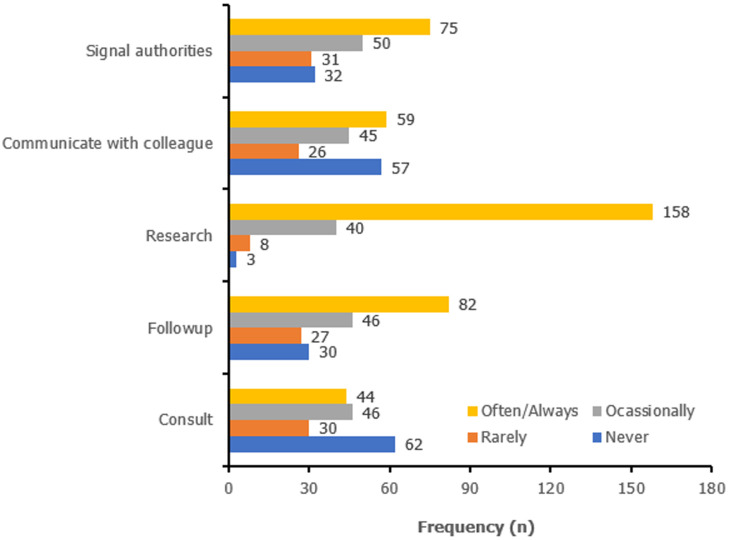
purpose of use of digital tools

**Reasons for and barriers to the use of digital devices:** health-related research (75.6%) was the most frequent reason why participants used digital devices, followed by patient follow-up (44.3%) and signalling public health authorities about potential threats (39.9%) as shown in Figure 2. Internet service was provided to 42.2% participants in health facilities with 48.8% of the participants qualifying the internet connection provided as good. Unreliable power supply was the major barrier to the use of digital health (59.9%) followed by recurrent technical failures (47%). The majority of participants (60.6%) had received some form of training in Information and Communication technology or Computer Sciences while 38.6% of the participants saw lack of training as a barrier to the use of digital health making it the third biggest barrier to the use of digital health.

We found that 39.4% of participants used digital health. Variables associated with the use of digital health were assessed using the Chi-squared test. Owning a laptop (OR = 2.1, CI, 1.18 - 3.78, p = 0.018), owning a tablet (OR = 3.63, CI, 1.08 - 12.20, p = 0.039), availability of internet connection in health facilities (OR = 1.77, CI, 1.02 - 3.07, p = 0.050) and receiving professional training in ICT/Computer Sciences (OR = 2.1, CI, 1.18 - 3.79, p = 0.016) were statistically significantly associated with use of digital health as shown on Table 1. After multivariable logistic regression analysis, owning a laptop (aOR = 1.98, CI, 1.01 - 3.86, p=0.046), having internet service provided in health facility (aOR = 1.99, CI, 1.05 - 3.79, p = 0.035) and receiving professional training in ICT/Computer Sciences (aOR = 2.04, CI, 1.06 - 3.93, p=0.033), remained independently associated with use of digital health (Table 2).

**Table 1:** predictor variables associated with the use of digital health technology

Factor	Total (N)	Uses digital health	Odds ratios	95% CI	p-value
**Age in years**					
≤ 40 years	161	67 (85.90%)	0.67	0.30 - 1.46	0.41
> 40 years	34	11 (14.10%)			
**Gender**					
Male	50	21 (25.30%)	1.17	0.61 – 2.24	0.65
Female	163	62 (74.70%)			
**Type of facility**					
Public	79	27 (32.14%)	1.41	0.79 – 2.50	0.31
Private	135	57 (67.86%)			
**Owns Smart Phone**					
Yes	191	78 (91.76%)	1.28	0.48 – 3.35	0.79
No	20	7 (8.24%)			
**Owns Laptop**					
Yes	71	37 (52.11%)	2.11	1.18 – 3.78	0.018
No	138	47 (47.89%)			
**Owns Tablet**					
Yes	13	9 (10.71%)	3.63	1.08 – 12.20	0.039
No	196	75 (89.29%)			
**Internet service provided in facility**					
Yes	92	44 (50.57%)	1.77	1.02 – 3.07	0.50
No	126	43 (49.43%)			
**Received professional training in ICT**					
Yes	129	61 (70.93%)	2.12	1.18 – 3.79	0.016
No	84	25 (29.07%)			
**Poor understanding of technology**					
Yes	57	18 (20.93%)	0.52	0.27 – 0.99	0.068
No	145	68 (79.07%)			
**Recurrent technical failures**					
Yes	94	47 (55.29%)	1.79	1.02 – 3.15	0.06
No	106	38 (44.71%)			

**Table 2:** multivariate logistic regression analysis of significant predictor variables and outcome variable “Uses digital health”

Factor	Adjusted Odds Ratios	95% CI	p-value
**Owns Laptop**			
Yes/No	1.98	1.01 – 3.86	**0.046**
**Owns Tablet**			
Yes/No	3.18	0.68 – 14.74	0.14
**Internet service provided in facility**			
Yes/No	1.99	1.05 – 3.79	**0.035**
**Received professional training in ICT**			
Yes/No	2.04	1.06 – 3.93	**0.033**
**Poor understanding of technology**			
Yes/No	0.42	0.20 – 0.88	0.02
**Recurrent technical failures**			
Yes/No	1.98	1.04 – 3.74	0.037

## Discussion

This study aimed at assessing the level of use of digital health among healthcare workers in Buea and Tiko health districts and identifying factors associated with its use. The prevalence of use of digital health among participants was 39.4% with owning a laptop, having internet service provided in the health facility and receiving formal training in ICT/Computer Sciences being significantly associated with the use of digital health after multivariable logistic regression.

Participants between the age range of 19 - 48 years accounted for 94.9% of the study population with a modal age of 25 years. This age group has been reported in the U.S.A. and other countries to be more likely to use technology with higher rates of acceptance of new technology [12-15]. Most participants reported that the most available digital devices in healthcare facilities were fixed phones, desktops and feature phones while modern devices such as the smartphone, laptop, tablets and cameras were rare. The decline in the use of devices like the desktop computer to access internet will result to an increase reluctance of healthcare workers in these districts to access internet-based digital technologies [16]. Healthcare workers who depend solely on devices provided by the facility for their work would be severely handicapped by the limited range of services that these devices offer [17]. The unavailability of modern devices in health facilities thus makes DHIs hard to implement.

A majority of participants owned a smartphone. Flynn *et al*. reported similar trends in the U.S.A. in 2018 where 98% of nurses were found to use a smartphone in the acute care setting [18] but differed significantly from a study done by Zurovac *et al*. in Kenya in 2012 where only 1.7% of healthcare workers were found to have smartphones [19]. The fact that most participants owned smartphones means a potential for rapid uptake and easy integration of mhealth technologies. The smartphone, laptop and desktop were found to be extremely/very useful by most participants for their healthcare practice. There is a new trend towards the use of portable devices with faster processing abilities as opposed to other less movable devices [20,21]. Participants who owned a laptop and a tablet were more likely to use digital health. When device utility was stratified by profession, we noted preferences as follows: doctors preferred tablets, nurses preferred smartphones while laboratory attendants and pharmacists preferred laptops for their healthcare work. This preference should guide investment in purchasing digital devices to facilitate the integration of the device in the workflow and hence the uptake of any related DHIs implemented through it.

EHR use was low with only 17.8% of participants reporting rare or occasional access and 13.67% having frequent access. HIV nurses and counsellors followed by laboratory technicians had the highest access to EHR, similar to results found by Akanbi *et al*. which showed that EHR use in sub-Saharan Africa was mostly by HIV-related health centres [22]. The fact that these systems were already in use in HIV programs and in laboratories made it relatively easier for them to be implemented in other areas of clinical the clinical setting. Despite the overall low penetration of EHR demonstrated by our study, the increasing uptake of EHR, is consistent with that found in other countries of sub-Saharan Africa [23]. Use of digital tools like MS Excel, MS Word and MS PowerPoint for work-related purposes was still low. DHIs interventions, that participants made comparatively more use of were research and diagnosing applications. Health-related research was the most common reason why participants used digital health. This is similar to conclusions by Mosa *et al*. whereby disease diagnosis applications were found to be most useful to healthcare professionals and by Moore *et al*. in an online survey which involved nurses and doctors practising in the UK [20,23]. These findings present opportunity for the implementation of Clinical Decision Support Systems.

This study found that more than 50% of participants did not have access to internet service at their healthcare facility. Less than half of participants who reported having access to internet said that the connection was good. Internet access plays a key role in the ability to implement and utilize the full range of digital health services hence the unavailability of good and stable internet access could hinder the ability to make use of digital health services. A majority of the participants were computer-literate hence should be able to use basic digital tools and software. This significant computer-literacy rate provides opportunity for the easy uptake of digital health, increasing the odds of its use by 2.12. This will reduce the time and expenditure required to train healthcare workers on the use of any DHIs implemented.

The three most frequent barriers that participants encountered in their use of digital health were unreliable power supply, recurrent technical failures like devices frequently getting bad and lack of training on the use of digital technologies. The findings in our study are consistent with those observed in a survey by the African Strategies for Health project [24]. Other studies also report similar findings [25,26]. Infrastructure, stable power supply, reliable internet connection, strong telecommunication systems and good legislation, among others are a prelude to a proper functioning of a digital healthcare system [11]. Without these conditions, digital health becomes harder to implement and when implemented, its full benefits cannot be exploited. A completely digitalized healthcare system, implemented in this context with no backup will hamper the workflow. The prevalence of use of digital health among participants was below average. Participants who owned a laptop, owned a tablet, had internet service provided in health facility and had received professional training in ICT/Computer Sciences were more likely to use digital health than participants who did not.

**Study limitations:** there is no standard definition of a person who uses digital health so the definition used in this study might not reflect the true amount of use of digital health in the population. Political insecurity did not permit access to all health areas under the health districts selected especially those in rural areas most affected by the ongoing crisis. We cannot establish causality of the observed associations as this was only a cross-sectional study.

## Conclusion

This study shows a low level of use of digital health by healthcare workers. Providing newer devices, internet connection in health facilities and training in ICT for healthcare workers could improve its uptake.

### What is known about this topic

- *There's an increase transition from paper-based to digital information systems in LMICs;*
- *mHealth and other DHIs has a potential to transform health service delivery in the developing world*.

### What this study adds

- *A prevalence of use of digital health among healthcare workers in two districts of 39.4%;*
- *Lack of training in ICT and lack of internet services in healthcare facilities pose a major barrier to the use of digital health technologies;*
- *Providing newer and portable devices with faster processing speeds could significantly increase the use of digital health by healthcare workers*.
